# Dignity of the nurse: A hermeneutic literature review

**DOI:** 10.1177/09697330261428626

**Published:** 2026-02-24

**Authors:** Yvonne Combrinck, Neltjie C. van Wyk

**Affiliations:** 1 Department of Nursing Science, 56410 University of Pretoria, Pretoria, South Africa

**Keywords:** dignity, professional dignity, nurse dignity, professional worth, self-worth, self-respect

## Abstract

The dignity of the nurse is a value that remains to be fully embedded in the nursing profession. It is a hidden concept that nurses tend to overlook. Regard for the dignity of the nurse is crucial because it enables nurses to fulfil their duties to the best of their potential. This literature review aimed to explore and better understand the meaning of the dignity of the nurse as it appears in education and nursing practice. A hermeneutic approach underpinned the methodology in the search, acquisition, and analysis of 16 studies. Dignity of the nurse as conceptualised in this review resulted in answering the following questions: in what ways is it defined; in what context does it appear; how is it sensed, and why does it matter? The review results confirmed the impact of dignity and indignity encounters on nurses. It is crucial to prioritise the dignity of the nurse as a value of equal importance in nursing.

## Introduction

In this article, the authors offer a hermeneutic interpretation of several noteworthy concepts concerning the dignity of the nurse. Nurses come into the world as human beings first. They possess human dignity that cannot be lost or taken away.^1^ Nurses also hold dignity in their capacity as professionals in the nursing profession. This dignity is both delicate and acquired, and may be preserved or lost at any time during nursing encounters in everyday work life. Nurses can never separate being human from being a nurse. It forms part of being a professional person.^2^

## Background

Dignity of the nurse is a professional value^3^ that should be honoured in equal standing in nursing.^4^ It is an evolving concept which has only come to the fore in the last two decades. Its constructs are rooted in nurses’ self and professional worth^2^ as experienced in their work environments. Dignity of the nurse is an important value in nursing because nurses’ potential to provide quality nursing care is dependent on their encounters of dignity and indignity.^5^ Nurses are, by nature, more inclined towards prioritising the dignity of patients. Their nursing values are aligned to honour patient-centred care and to put their patients’ needs and dignity above their own.^6^ Regard for the dignity of the patient could never be at the cost of the dignity of the nurse. The dignity of the nurse is an inherent and professional right.^7,8^

Nursing ethical codes acknowledge the dignity of the nurse as a self-regarding duty. It emphasises nurses’ responsibility to care for themselves and protect and preserve their individual character, personal health, and well-being. It also places responsibility on organisations to provide conducive nursing environments where nurses are supported through effective education and management. The nursing ethical codes honour nurses’ dignity and worth and confirm their regard for themselves in equal standing to that of their patients.^7–9^ Nurses are rightfully entitled to the same worth and dignity bestowed on patients:

‘The nurse has moral duties to self as a person of inherent dignity and worth including an expectation of a safe place to work that fosters flourishing, authenticity of self at work, and self-respect through integrity and professional competence’.^7prov5^

A growing body of knowledge to better understand the dignity of the nurse in various healthcare settings and education is evident in the literature. Research in exploring nurses’ dignity experiences originated in Italy, followed by further research in South Africa, Finland, Norway, and Iran. Various perspectives were gained in hospital, community, and palliative healthcare settings.^5,10,11^ The research focused on the experiences affecting nurse dignity positively,^12^ or negatively,^13,14^ including strategies for preserving nurses’ professional dignity.^4^ An interest in exploring the dignity of the nurse in nursing education was also evident.^15–18^ The development, validation, and implementation of nursing and workplace professional dignity scales within societal contexts contributed further to the evolving body of knowledge. It provided healthcare organisations with valuable tools to measure the dignity of nurses and action strategies for preserving and enhancing nurse dignity.^19–21^

The dignity of the patient is a significant value in nursing that has been thoroughly examined within the profession. However, the dignity of the nurse has received considerably less attention.^3^ To our knowledge, there is currently no review that conceptualises the growing body of research on nurses’ dignity. This study aimed to explore and describe the dignity of the nurse by examining its definitions, context, sensing, and importance. The goal is to enhance understanding of how this concept is reflected in nursing practice and education.^22^

## Methods

### Design

A literature review is a recognised research method for generating new knowledge from an existing body of previously published research to gain insights into concepts and phenomena in the world we live in. It involves acquiring relevant publications through searches of large databases, followed by inquiry, interpretation, and critical assessment to better understand the text and its context in new and imaginative ways.^23^ The authors adopted a hermeneutic approach as a methodology in this literature review to meaningfully engage with the acquired literature. According to Gadamer,^24^ researchers actively enter into dialogue with the text at hand through what is called ‘the fusion of horizons’.^24p189^ When the ‘horizons’ of the researcher and the text come together, seeing anew broadens to make way for the interpretation of the text, in synergy with pre-knowledge and pre-understanding. This occurs through a hermeneutic circle, moving between the individual text (parts) and the whole of the literature (context).^23,24^

The Boell and Cecez-Kecmanovic’s^23^ hermeneutic structure for literature reviews was followed, which comprises two hermeneutic circles. The search and acquisition circle included the steps of searching, sorting, selecting, acquiring, and reading. The circle of analysis and interpretation were guided by analytic reading, mapping, classifying, and critical assessment. The authors were cognisant of their pre-understanding while striving to be open to the ‘otherness’ of the reviewed literature, to see the concept of dignity and its constructs anew.^24^

#### Searching for literature

During the search, publications were sourced from the databases of CINAHL, PubMed, Medline, and EBSCOhost. Key terms such as “professional dignity,” “nurse* dignity,” “nurse*,” “dignity,” “workplace dignity,” and “dignity of the nurse” with the Boolean operators AND, OR, and NOT were utilised. Google Scholar and Web of Science served as complementary search tools in combination with the referenced literature in the sourced publications.

#### Sorting and selecting

Inclusion and exclusion criteria were used to sort and select publications based on their relevance to the dignity of the nurse. Qualitative studies were considered in which the dignity of the nurse emerged as a theme, with a focus on nurses’ perceived experiences of nurse dignity in nursing practice and education. Publications related to conceptualisations or synthesis of the dignity of the nurse and its definitions were also included. There was no restriction on the year of publication. Commentaries, letters, and publications not related to the dignity of the nurse, such as dignity in care, workplace and human dignity, or dignity of healthcare workers that are not nurses, were excluded.

#### Acquiring and reading

**Figure 1.**
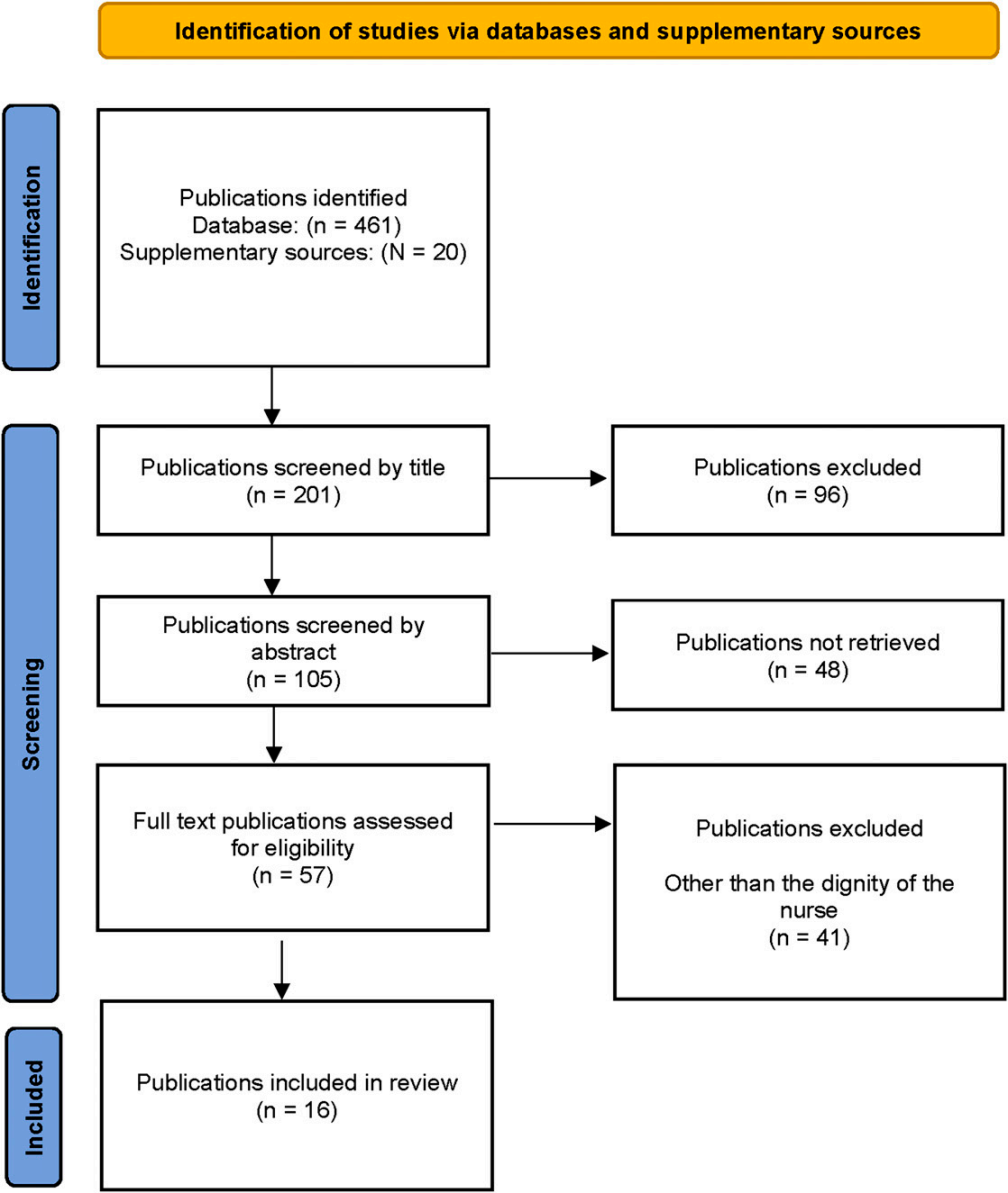
PRISMA flow diagram for data collection and study selection (adapted from Page et al. 2020). For more information, visit: https://www.prisma-statement.org/. *From:* Page MJ, McKenzie JE, Bossuyt PM, Boutron I, Hoffmann TC, Mulrow CD et al. The PRISMA 2020 statement: an updated guideline for reporting systematic reviews. BMJ 2021;372:n71. https://doi.org/10.1136/bmj.n71.

The sourced publications were first sorted and screened by title. Reading of the abstracts followed. Peer-reviewed publications regarding nurses and their dignity in nursing practice and education were included. Publications with a focus on dignity in care and patient dignity were excluded. Duplications were eliminated. This resulted in 57 full-text publications eligible for reading. The focus was on the inclusion criteria of nurses and their dignity as it appears in nursing practice and education. Sixteen publications between 2012 and 2024 were selected for inclusion in the review. A quality appraisal of the selected publications was conducted using the Critical Appraisal Skills Programme (CASP) UK (n.d.) to assess the papers’ trustworthiness, relevance, and results.^25^ The information about the searched and acquired publications is provided in Figure 1.

#### Analysis and interpretation

The analysis and interpretation circle was entered with a sense of curiosity to establish what was known regarding the dignity of the nurse. The authors engaged with the literature as a dialogical encounter, drawing on their pre-understanding. The aim was a ‘fusion of horizons’^24p189^ between the authors and the publications at hand, to see anew with a broadened horizon. The publications were classified by author, date, country, purpose, methodology, and findings for a summarised oriental perspective of the acquired literature (refer to Table 1). Through analytical reading of each publication, the content was sensed both as per individual publication and as a complete body of knowledge. Side notes were documented as a conceptual map for interpreting, understanding, and classifying ideas and literature findings. These were critically assessed while staying open to emerging concepts and a new understanding of the dignity of the nurse as conceptualised through its definitions, appearance, sensing, and significance.

**Table 1.** Details of included research.

Author and country	Purpose	Methodology and discipline	Major findings
Abbasi et al. (2023). Iran	Aimed to explore nurses’ perceived threats to their professional dignity.	A qualitative study with 15 in-depth semi-structured interviews.	Threats to nurses’ professional dignity were related to professional and organisational factors such as violence, nurse incompetence, inappropriate nurse behaviour, and novice nurse placement.
Combrinck et al. (2020). South Africa	To explore and describe factors affecting nurses’ professional dignity in private hospitals.	Phenomenological study, in-depth individual interviews. 11 nurses	Professional dignity was influenced by pride, support, and appreciation. Complex private healthcare and humiliation influenced professional dignity adversely. Professional dignity was seen in view of self and others.
Combrinck et al. (2022). South Africa	Develop and refine strategies to preserve the professional dignity of nurses.	Qualitative with two focus group discussions. 16 healthcare professionals.	Six strategies were developed for preserving nurses’ professional dignity. These included preserving nurses’ pride, professional roles, and desire to prioritise patient care. Strategies for coping with humiliating and complex healthcare experiences were also included.
Froneman et al. (2019). South Africa	To explore and describe the enhancement of the professional dignity of midwives in a hospital.	Phenomenological study with in-depth individual interviews. 15 midwives.	Dignity-enhancing factors were skills acknowledgement and appreciation, midwife autonomy, team harmony and inclusion, and management support and care.
Gustafsson et al. (2017). Sweden	Aimed to describe violations of nurses’ professional dignity in psychiatric facilities.	A hermeneutic study with 18 group discussions over 9 months. 10 nurses in two psychiatric facilities.	Nurse participants experienced violations of their dignity from caregivers and patients. Some caregivers regarded themselves as of a higher rank than the nurses and did not share critical treatment information. Nurses were, at times, forced to perform repulsive acts. Patients called nurses humiliating names and were, at times, physically abusive towards the nurses.
Khademi et al. (2012). Tehran	Explore and understand the violations of nurses’ professional dignity in two hospitals.	A qualitative study with 26 unstructured interviews in two hospitals. 15 nurses.	Violations revealed: Irreverence with abuse, lack of recognition for autonomy, skills, and the value of nurses’ contributions.
Sabatino et al. (2014).	To create a tentative model for the concept of professional dignity.	A meta-synthesis of qualitative research	Nursing professional dignity constitutes the characteristics of human beings and workplace elements.
Sabatino et al. (2016). Italy	To describe the perceived professional dignity of nurses in internal medicine and surgery departments in hospitals.	A qualitative study with 20 focus group discussions. 124 nurses working in internal medicine and surgery departments in 15 public health organisations.	Dignity is perceived within the self and others. Human dignity was seen as part of professional dignity. Relations with physicians, nurses, and support workers influenced professional dignity.
Stievano et al. (2012). Italy	To explore and describe nursing professional dignity in relationships with health professionals in clinical and community workplaces.	A qualitative study with 11 focus group discussions. 72 nurses working in hospitals and community settings.	Professional dignity is achieved better in community nursing than in hospitals through autonomy and teamwork across disciplines. Recognition of dignity beyond professional roles constitutes nurses’ intrinsic dignity as an expression of humanity.
Stievano et al. (2019). Italy	To present nursing’s professional dignity as experienced in palliative care.	A qualitative study with 12 focus group discussions. 69 nurses working in hospices and home care.	Main themes included intrinsic dignity, professional relationships, professionalism, and patient and significant others’ relationships.
Stievano et al. (2022). Finland	Aimed to describe community health nurses’ perceptions of nursing’s professional dignity.	Qualitative study with semi-structured focus group interviews. 27 public health nurses in primary healthcare.	Professional dignity was expressed as self-respect for one’s competencies and skills and trust from those to whom services have been rendered. It was also linked to interprofessional relations and community standing.
Stikholmen et al. (2022a). Norway	To understand nursing students’ experiences of their dignity in relation to their supervisors.	Hermeneutic study with narratives and qualitative interviews. 19 nursing students.	Student dignity was experienced in supervisor encounters, and their student affirmation was achieved through acknowledgement. It impacted student courage and presence in the moment.
Stikholmen et al. (2022b). Norway	To understand how nursing students discover and acquire their dignity.	Hermeneutic study with qualitative interviews. 19 nursing students.	Nursing students came to know their dignity through introspection and interaction with others. This included experiences of vulnerability, pride, and shame. Dignity consciousness developed from dignity and indignity encounters.
Stikholmen et al. (2024). Norway	To describe nursing educators in promoting dignity and the experiences thereof.	Hermeneutic study with five focus group discussions. 21 nurse educators.	Promoting dignity in nursing education was realised through enhancing the dignity of the nursing profession and the students, and maintaining dignity in challenging situations.
Tehranineshat et al. (2022). Iran	Exploring and describing nursing students’ dignity in clinical settings.	Qualitative descriptive study with semi-structured in-depth interviews. 21 nursing students.	Dignity promoters were trusting relationships and developing professional identities.
Valizadeh al. (2018). Iran	To explore and describe threats to nurses’ professional dignity in relation to their wanting to leave clinical practice.	A qualitative study with 21 semi-structured interviews.	A lack of professional pride, an oppressive work environment, and a lack of appreciation and progressivism threatened nurses’ dignity.

## Findings

Dignity of the nurse as conceptualised in this review resulted in answering the following questions: in what way is it defined; in what context does it appear; how is it sensed, and why does it matter? The reviewed studies used the terms dignity and professional dignity of the nurse within the same context; henceforth, the term dignity will imply both professional dignity and dignity as such.

### ‘What?’ In what way is dignity of the nurse defined?

The dignity of the nurse is a value-based, multifaceted concept that encompasses the entirety of a nurse’s professional being.^2^ It is perceived within the social constructs of nurses’ standing in relation to themselves, others, and the work environment.^2,5^ It is reflected in nurses’ subjective opinions and perceptions of themselves, as expressed in their self-respect, self-worth, and self-esteem. It also comprehends nurses’ professional identity, worth, respect, autonomy, competencies, and integrity, as shaped by their work relationships and environment.^2^ The dignity of the nurse is also defined beyond merely recognising nursing professional roles to acknowledge the inherent dignity of every human being, including nurses themselves.^26^ Nurses’ standing in relation to themselves, others, and the work environment is inseparable from the inherent dignity of nurses. The constructs are interconnected and intertwined to present the dignity of the nurse in its completeness.^2^

### ‘Where?’ In what context did dignity of the nurse appear in various nursing settings?

Dignity of the nurse, as it appeared in various nursing settings, is discussed in this section.

#### ‘Dignity becoming’ in nursing students

Dignity evolved as ‘dignity becoming’ in the student participants. They discovered their dignity in fragile spaces, being at the ‘mercy’ of their educators, clinical supervisors, and nursing teams in nursing practice. They were continuously aware of their dignity being at risk in a practice environment with expected perfection, where ‘having a bad day’^16p198^ was not affordable. Their top priority was striving to live up to the expectations set by educators and clinical supervisors. They looked up to their educators for setting an example to follow.^16^ Their dignity was uncovered through trusting relations and professional identity. The student participants felt valued when they were trusted to care for their patients in a safe clinical environment free from fear. Taking accountability for acquiring skills and knowledge was fundamental to their dignity and self-respect. Trust in performing tasks allocated to them left the students with a sense of student autonomy. The student participants’ dignity was also dependent on receiving respect from others. It appeared in being treated politely and free from discrimination. When students were corrected in a private setting, away from patients, their self-esteem and confidence were preserved.^18^

#### A longing for excellence in nursing educators

Standing upright was at the core of the nursing educator participants’ dignity. They respected their mandate to uphold the profession’s standards and ensure competent students through learning in a dignity-promoting environment that accommodated students’ individual learning needs. Being a role model in portraying the values of the profession was a role that educators held close at heart. A student graduating without meeting standards was perceived as the ultimate form of indignity because a patient can never be compromised in such a way.^17^

#### Thriving as autonomous nurse practitioners in community care

Professional dignity was perceived as a prominent value in community nurses’ work environment. The nurse participants worked autonomously as part of a multi-professional team that valued their unique contributions. They felt trusted and appreciated by the community members who relied on them to provide direction in their health needs. They experienced dignity when their services were preferred by choice. The nurse participants believed people would recognise their profession’s dignity when they conducted themselves professionally and respectfully. Displaying competence and knowledge earned respect for themselves from patients and other health professionals.^11^ Their dignity was perceived as an achievement.^26^

#### Being at the mercy of demanding healthcare in acute care settings

Dignity in acute care revealed itself through a mixture of dignity and indignity encounters. As skilled independent practitioners, the nurse participants desired to give themselves to their full potential for the good of the patients they cared for. They felt unworthy in the eyes of their patients and themselves when hindered from utilising their knowledge, skills, and capabilities. Doctor superiority, workload, unsupportive management, and a lack of resources influenced dignity adversely.^12,13^ There were also moments when the nurse participants stood in awe of their perceived dignity. Nursing science applied in clinical practice, a successful resuscitation, a thank you from a manager, and seeing a patient leaving the hospital recovered and well, instilled a sense of professional dignity and pride in the nurses.^5,27^ Nurses in acute care cannot practice in isolation from others or their work environment. Exposure to a fast patient turnover, excessive paperwork, daily cost containment pressures, and disrespectful encounters left the nurse participants questioning themselves, their purpose, and their nursing calling.^5^

#### Respect for human life in vulnerable spaces in palliative care

Professional dignity was uncovered in the closeness with humanity in palliative care. With a deep awareness of the inherent dignity of people, respect for human life was central to the participants’ experience of professional dignity. Protecting patients’ dignity while staying professionally true to integrity was at times hindered by ethically challenged circumstances, which adversely affected professional dignity. There was a strong sense of working together in a team, and the participants were disappointed in some healthcare practitioners who did not uphold standards and respectful interactions in palliative care. Compromising patients while in a most vulnerable space affected the study participants and their professional dignity.^10^

### How? How is dignity of the nurse sensed?

#### Sensing dignity through humanity

The nurse participants were conscious of being human, irrespective of their professional nurse roles: ‘Before we are professionals we are people…’^27p284^ Their personal dignity was prioritised before their professional dignity.^10,26^ They desired to be treated with respect for their worthiness as unique individuals.^5,15,27^ Their dignity was realised when they were seen for the persons they were within their own social context.^17^ They perceived being a good nurse as a person of good character with sound ethical values.^16,26^ Their nursing actions were constituted by acknowledging that all people, including nurses, possess inherent dignity that cannot be lost because they are human beings.^10,17,26^ When managers and educators supported them during vulnerable times, they felt respected as human beings: ‘l felt like a human again…’^5p399^ Being addressed by their names was important.^15^ They did not want to be mere objects in their workplaces to get the job done.^5^ The study participants reflected on the impact of a demanding nursing environment. It affected them as human beings: ‘It has such a powerful effect on your total human being’.^5p399^ When they failed at performing a task well, they felt they failed as a person and not just as a nurse.^16^

‘It’s so personal, in a way. You use yourself. And when you fail, it is you as a human being that fails. [ …] So, when you use yourself, you become vulnerable to criticism. Because it’s you who’s not good enough’.^16p199^

‘The name is me, it’s my identity…Everyone can be a student, but only I am Natalie’.^15p1606^

‘The dignity exists because a human being is a source of dignity. I have got a sense of dignity, of honour, of being what I am…’^10p1638^

#### Sensing dignity through professional worth

The study participants sensed their dignity in standing professionally in nursing practice and education. They were conscious of being worthy of a profession they held in high esteem.^5,15,16^ A neat appearance and portraying themselves with grace and exceptional professional conduct were non-negotiable.^16,18^ Nurse educators were, in particular, aware of how they carry themselves: ‘So it’s about physical presence. How to enter a room. It’s about how you use your voice. It’s about how you use your gaze’.^17p115^ The study participants sensed dignity in executing their tasks masterfully, with their patients’ well-being at the centre of their calling in being good nurses.^5,15,16^ When nursing science unfolded with precision and became an effortless action, professional dignity and pride emerged in their highest form.^5,27^ Being knowledgeable, skilled, and clinically well prepared during their encounters with others earned them respect, trust, appreciation, and autonomy in their work environments.^5,11,15–18,26^ The study participants experienced a deep sense of dignity in their professional standing with others. They wanted to be trusted to carry out their tasks to a high standard. When they were ‘seen’ in their professional encounters with others, they felt worthy above and beyond: ‘I felt seen…She made me feel visible…’^15p1606^

‘We are a default value for all Finns – it is a sign of professional dignity’.^11p8^

‘But that for me, spoke such professional dignity, made me feel like what I’ve been trained and taught, through my ethos, through social science and just my general anatomy and physiology put everything into perspective when it comes to patient care and made it seem like this is an effortless job’.^5p398^

#### Sensing dignity through consciousness

Although the study participants reflected on the dignity of the nurse as a hidden concept not often thought about in everyday nursing, they knew when their dignity was honoured or threatened.^11^ Dignity consciousness constituted a sense of dignity and the need to preserve and protect it. Dignity was described as bodily sensed moments of being proud, respected, and seen, or shamed, humiliated, and overseen.^16^ These moments led to being conscious of future dignity experiences and how they might present.^28^ The study participants were therefore cautiously aware when exposed to dignity-threatening encounters to respond in protection of their dignity ‘…I kept completely quiet and tried to be polite…I didn’t want to be humiliated…’^18p9^ The study participants were also cognisant of being instrumental in promoting dignity,^17^ as well as witnessing others’ dignity being at stake.^5,13^ According to them, dignity was sensed through words and behaviour in interaction.^11^ It was perceived to be present in every action and gesture of a person.^26^

‘It’s so difficult to define dignity, but I know when something is dignified. I also recognize when dignity is lacking. I can give many examples of when I feel dignified and when I do not feel dignified’.^16p200^

‘If you have first experienced that feeling of having no dignity, you become aware of it. I am very aware of it now’.^16p200^

### Why? Why does dignity of the nurse matter?

#### Dignity of the nurse in awe

The study participants reflected on moments of standing in awe of their dignity. It instilled a sense of worth, pride, motivation, and energy in their work. Such experiences contributed to their well-being, quality of life, and self-esteem.^15^ They reflected on being confident and fully engaged in their learning and nursing activities to the full of their potential: ‘You get a boost (…) Dare to go in, dare to be. To stand there, true. And be there’.^15p1608^ Being safe in the moment contributed to mindful interaction, quality dignified nursing care,^2,5,15,26^ and confidence in asserting nurses’ respect and professional dignity^5,11^: ‘If you have your own dignity, you make yourself to be respected, nobody can take the dignity away from you’.^26p348^

#### Indignity of the nurse

The study participants shared experiences of indignity affecting their work, professional standing, worth, and well-being. They felt neglected and undervalued following exposure to humiliation, belittling, managerial and medical superiority. Being undermined in their work and a profession they took pride in injured their professional identification and self-esteem.^5,13,14,18,29^ They felt powerless and voiceless and considered leaving their workplace or even the nursing profession altogether.^2,12–15,18,29^ The study participants reflected on experiences of indignity affecting their performance and work output. They described falling behind and losing confidence, motivation, and interest in their work. Indignity encounters resulted in biased care and a lack of quality patient care. In extreme circumstances, it led to mismanagement of patients and at times serious adverse consequences^5,12–15,29^: ‘You become frustrated…you start mismanaging…when you start mismanaging…patients start collapsing…patients die’.^12p1068^

Exposure to indignity experiences also affected psychological and physical well-being. The participants felt overwhelmed, helpless, and hopeless, as if the world were falling apart. There were experiences of depression, anxiety and isolation, and ‘being in a bubble’ for themselves. Another study participant described going in an ‘autopilot’ mode. Some experiences were physical and presented bodily, such as being nauseated and feeling sick to the extent of going off duty. Participants in a psychiatric setting blamed themselves for allowing a perceived dignity violation in circumstances they had no control over. They would then shut down entirely in the moment.^5,15,28^

‘You turn off everything. You cannot stand it… when you hear those screams that cut like a knife inside you. You think, what the hell am I doing? You try to shut down, to not hear it anymore. You almost become robotic. And I would refuse immediately if I had a choice. I felt so bad, could not sleep for many nights after that. As a matter of fact, I am still having nightmares about it’.^28p139^

## Discussion

### In what ways is dignity of the nurse defined?

Although the concept of dignity is vague, it has been studied since the Roman Antiquity when it indicated the position of individuals in society. In the context of this article, the dignity of nurses applies to their position in the healthcare society as well as in the society-at-large. What position do they hold in relationship to colleagues and the society that they serve? According to Cicero (106-42 BC), dignity refers to the public’s recognition of a person’s social position. Dignity is not established by oneself, is permanent, and is determined by the opinion of the group to which the person belongs. It is the collective decision of the group, it often relates to positions of power, and it is valuable for order in the group.^30^ When nurses are considered as just members and never as leaders of healthcare teams, they are perceived as inferior to leaders. Nurses’ capabilities, no matter how advanced, have in this case no impact on their dignity. Such perspectives of nurses’ dignity do not align with the International Council of Nurses’ definition of nursing that emphasises the management and leadership roles of nurses. Nurses work autonomously and collaboratively across healthcare settings to improve health.^31^

### How is dignity of the nurse sensed?

Ricoeur (1913-2005) introduced another definition of dignity. According to him, persons’ perceptions of themselves as dignified beings should be acknowledged. Dignity is dynamic and multifaceted, influenced by persons’ self-esteem and self-respect, and results in recognition by others. Self-esteem, according to Ricoeur, is the basic form of human dignity and represents persons’ feelings of being someone as well as the wish to be respected by other people. It results from the interaction between ‘being’ and ‘ought to’ and represents the desire to ‘exit’ rather than to merely ‘be’. The desire to ‘exit as respected persons’ is associated with the vision of what good lives entail. The same yardstick is used to ‘valuing selves’ than ‘valuing others’.^32,33^ Nurses’ self-esteem can therefore be described as the outcome of their own ‘self-valuing’ narratives. The values that persons apply to measure others’ values apply to the measurement of their own values.^34^ Nurses who do not value the input that they make to the recuperation of patients cannot expect others to value their profession. On the other hand, when healthcare team members and patients cause them to feel undignified, nurses’ self-esteem gets jeopardised. They are unfortunately at times exposed to rudeness from members of the healthcare team, patients, and their family members due to stressful circumstances that are common in hospitals and more specifically in emergency and palliative care settings. When life-threatening situations occur, people tend to blame others for poor care outcomes. When nurses are blamed when patients perceive their care as substandard, their dignity is at risk. According to De Beer, Rawas, and Beheri (2024), respect for nurses’ dignity does not only lead to their well-being but may also impact positively on their performance in delivering quality patient care. When the opposite happens, nurses may feel demotivated to keep on doing their utmost best in patient care.^19^

### In what context does dignity of the nurse appear?

According to Ricoeur^35^ (1992), self-esteem is transformed into self-respect when values are operationalised into behaviour. The feelings and wishes of the self-esteem get transposed into duties judged by morality. Self-respect, according to Ricoeur, is self-esteem expressed in the context of norms dictating what is morally acceptable. ‘Valuing oneself and others’ with the same yardstick requires persons to judge themselves with the same norms as they use for others with the aim of supporting equality in respecting dignity.^32,36^ The public respects the nursing profession when nurses display behaviour associated with deontological ethics. Nurses’ duty-based ethical behaviour is not only expected by others but also by themselves. Nurses perceive their duty to respect and enhance patients’ well-being as their utmost responsibility. Patients are ill, experience pain, feel vulnerable in the unfamiliar settings of hospitals and clinics, and are very dependent on nurses’ sensitivity for their needs to regain their independency. When nurses react through valuing in the same manner as they value themselves as dignified human beings, professional values are operationalised into ethical duty-based nursing care. In the dignified care of patients, nurses’ self-respect is enhanced. According to the teaching of Ricoeur, self-respect requires persons to be ‘commanders and the commanded’ at the same time.^32^ Nurses set the criteria for duty-based ethical behaviour and have to see to it that their practice meets the criteria.

### Why does dignity of the nurse matter?

Self-esteem and self-respect enable ‘recognition’ as an act through which the ‘selves’ of persons get acknowledged equal. It is according to Ricoeur^37^ the final form of human dignity and implies that persons’ ‘selves’ are distinguished from that of others, recognised as unique, and worth equal respect. Differences are acknowledged, but equality is appreciated.^32,38^ Healthcare professional persons differ in their roles within the team, but all members deserve equal respect. Differences should be acknowledged, but mutual respect should be secured. One professional group is not more important than others in the healthcare team. The professional dignity of all team members should be enhanced. It is dynamic and can be strengthened by individual members and by the team. Dignified healthcare teams deliver quality care to the benefit of patients.

### Limitations of the study

This literature review was based on the acquired literature through an existing database and may not be a reflection of the entirety of the subject matter. The review focused on the dignity of the nurse through its definitions, context, sensing, and significance. Subject matter related to human and workplace dignity in general was not reflected upon. Although the authors sought to remain open to new and imaginative ways of understanding the dignity of the nurse, their prior knowledge and understanding may still have constrained the outcomes of the findings.

## Conclusion

Understanding the dignity of the nurse and its meaning, as presented in this paper, constitutes the foundation for embracing nurse dignity as a significant value in the nursing profession. It provides nurses with the means to confidently and respectfully assert themselves in their professional standing in their work environments. Some nurses found themselves engaged in an environment of disregard and disrespect, with resignation as their only perceived escape. It is a consequence that a profession, thin on resources, cannot afford. Respect for the dignity of the nurse supports nurses’ motivation and engagement in providing quality nursing to their full potential. It fosters positive, dignity-promoting cultures in which nurses thrive, and patients benefit from the best possible care.

The dignity of the nurse is a complex, multidimensional concept deeply rooted in the self and professional worth of nurses. It is sensed in their humanity, within unique individual contexts, and in them being instrumental through nursing science in providing excellence in the service of humankind. Self-esteem and self-respect are significant constructs of the dignity of the nurse. Experiences of dignity and indignity encounters contribute to a sense of dignity consciousness and a cautiousness for protecting and preserving dignity. Further studies are necessary to embed the concept of the dignity of the nurse as a valued phenomenon. The dignity of the nurse deserves the same standing as dignity in care in nursing practice and education.
